# Mechanism of worsening diabetic retinopathy with rapid lowering of blood glucose: the synergistic hypothesis

**DOI:** 10.1186/s12902-017-0213-3

**Published:** 2017-10-10

**Authors:** Ahmadou M. Jingi, Aurel T. Tankeu, Narcisse Assene Ateba, Jean Jacques Noubiap

**Affiliations:** 1 Department of Internal Medicine and Specialties, Faculty of Medicine and Biomedical Sciences, Yaoundé, Cameroon; 2 National Obesity Center, Yaoundé Central Hospital, Yaoundé, Cameroon; 3 Department of Medicine, University of Cape Town and Groote Schuur Hospital, Cape Town, 7925 South Africa

**Keywords:** Diabetic retinopathy, Insulin, Vascular endothelial growth factor

## Abstract

Insulin treatment has been associated with a paradoxical worsening of diabetes retinopathy since many years in European cohorts. Recently, this issue has been stressed by some studies conducted in other parts of the world. However, the mechanism underlying such evolution is not well understood. An osmotic theory has been evocated but failed to explain the clinical features of the disease. Considering recent findings from basic and clinical research, we discuss the possibility of a synergistic hypothesis based on the simultaneous action of insulin and vascular endothelial growth factor on eye blood vessels. We postulate that exogenous insulin could act synergistically with the vascular endothelial growth factor expressed by ischemic retina so as to trigger vascular proliferation and the worsening of diabetes retinopathy.

## Background

Diabetes mellitus is a chronic disease associated with significant morbidity and mortality owing to its multiple micro and macrovascular complications [1, 2]. Diabetes mellitus has reached epidemic proportions with the greatest impact in low income countries where it remains underdiagnosed, under investigated, and undertreated [3–5]. The epidemic rise in diabetes mellitus poses significant public health and socioeconomic challenges through diabetic complications affecting different organs with various impact. The eye is the most commonly affected organ in both type 1 and type 2 diabetes. [6]. Diabetic retinopathy is the most serious and commonest ocular complication associated with T2DM and one of the leading causes of secondary blindness worldwide [3]. Its prevalence has been reported to range from 15.3% to 42.4% in different studies [1]. Diabetic retinopathy is a disease characterized by microvascular alterations progressively leading to retinal ischemia, retinal hyper-permeability, retinal neovascularization, and macular edema. If left untreated patients with diabetic retinopathy can suffer severe visual loss [7]. In developed countries, diabetic retinopathy constitutes the leading cause of blindness in the working age population [8]. Different risk and progression factors for diabetic retinopathy have been documented and classified as modifiable risk factors (blood glucose, blood pressure, serum lipids, and smoking) and non-modifiable risk factors (duration, age, genetic predisposition, and ethnicity) [9]. However, recent literature has clearly demonstrated an incremented risk of diabetic retinopathy with regard to insulin treatment [1, 10–12]. Nevertheless, the mechanism underlying this paradoxical association is not well understood. Here we revisit evidence on the association between diabetic retinopathy and insulin use, and we propose a novel hypothesis to support this relationship.

## Insulin use and early worsening of diabetes retinopathy: Paradoxical report with growing evidence

It is established that diabetic retinopathy and related complications are strongly associated with the presence of chronic hyperglycemia, and results of almost all randomized trials are consistent with the fact that early and intensive glycemic control reduce both the onset and the progression of this condition [13–20]. This tight glucose control is better achieved with insulin therapy compared to oral antidiabetic agents as reported by major trials [19–21]. These findings along with the benefits of insulin on other vascular complications support the importance of tight glycemic control with insulin early in the course of diabetes [22]. However, tight glycemic control with insulin is associated with recurrent hypoglycemic episodes, and the risk of early worsening of diabetic retinopathy. This later has been reported in the Diabetes Control and Complications Trial (DCCT) where this occurred in 13.1% of the intensive group (using intensive insulin therapy) versus 7.6% of the conventional treatment group (using conventional insulin regime). However, this effect was reversed by 18 months, and no case of early worsening resulted in serious visual loss [19]. Similar finding were later reported by Wang et al. in a meta-analysis who found that the risk of diabetic retinopathy progression tends to increase after 6 to 12 months of intensive therapy with insulin (odds ratio: 2.11; 95% confidence interval: 0.54 to 8.31) compared to conventional treatment. Yet, this increase in diabetic retinopathy risk was also reversed after 2 years of intensive treatment with insulin. Recently, data on this paradoxical relationship have increased, with reports from others parts of the world [2, 11, 12]. This has been reported in both cross-sectional and longitudinal studies [23–25]. Further evidence drawn from more recent systematic review and meta-analysis support previous findings and highlight the early worsening effect of insulin on the progression of diabetic retinopathy [1, 10, 26].

## The role of osmotic force theory: Basis and limits

In view of the difficulty in explaining the paradoxical aggravation of diabetic retinopathy at the initiation of insulin therapy, osmotic theory was proposed based on physiological approach, and rapidly spread despite the few available arguments to illustrate this hypothesis [27]. This theory is based on the fact that glucose is an osmotically active molecule and can influence water movements according to the equation: Osmolarity = 2 (Na^+^ + K^+^) + Glucose + Urea. Therefore, an important change in glucose concentration in a given milieu can modify the osmotic pressure and act on water retention. To explain the early worsening of diabetic retinopathy with intensive treatment, it has been suggested that the rapid drop in plasma glucose concentration obtained with intensive and aggressive glucose lowering agents lowers the intravascular osmotic pressure. This creates an osmotic gradient between extracellular and intravascular compartment in favor of the interstitium. Water then moves from the higher osmotic pressure level (interstitium) to the lower osmotic pressure level represented here by vessels. This is more marked in the small vessels of the eyes, which are more sensitive to water retention. However, in spite of its logical basis, this theory remains limited and cannot explain major changes in diabetes retinopathy as well as the increase in this paradoxical worsening of diabetic retinopathy with insulin compared to oral anti-diabetic agents.

First of all, the osmotic drive force of glucose could plausibly support the occurrence of retinal edematous changes, but cannot explain the appearance of neo-vessels and other retinal features associated with diabetic retinopathy. In addition, it is worth noting that a rapid reduction of blood glucose by up to 4 g/l will not result to a significant change in serum osmolality than a change in the sodium concentration by 4 mEq/l according to the osmolarity equation mentioned above. This suggests that the osmotic drive depends more on change in sodium than change in glucose concentration. On the other hand, this theory cannot explain the higher incidence of diabetic retinopathy in patients on insulin rather as compared to those on oral anti-diabetic agents.

## A synergistic hypothesis could explained both the mechanism and clinical features of early worsening of diabetes retinopathy

Previous reports on the increased incidence and worsening diabetic retinopathy with insulin compared with oral anti-diabetic agents have led to the hypothesis that insulin is probably related to the occurrence of this paradoxical early worsening in diabetic retinopathy with rapid lowering of blood pressure. But the exact mechanism underlying this report is not well understood. Here we propose the synergistic hypothesis based on the simultaneous action of insulin and the vascular endothelial growth factor (VEGF) on eye blood vessels. Our theory is based on the fact that high dose of exogenous insulin could act synergistically with VEGF expressed by ischemic retina so as to trigger vascular proliferation and worsening of diabetic retinopathy. This explains the role of insulin, the different clinical features found on retinal examination, why diabetic retinopathy is more pronounced in individuals with pre-existing retinal lesions (worsening) and rare in individuals without any pre-existing retinal abnormality. This is based on evidence from basic science, epidemiological and interventional diabetes therapeutic studies [2, 3, 28], and could have important therapeutic implications.

## What do we learn from basic science research on the topic?

Insulin has been known for many years as an anabolic hormone necessary for growth. Experiences showed that growth hormone and insulin probably act together for an optimal growth [28]. For instance, in rats, when the pituitary gland and the pancreas have been removed, the administration of growth hormone or insulin alone does not produce any significant effect on growth. The concomitant administration of growth hormone and insulin results in an exponential growth pattern [28]. This provided evidence that insulin and growth hormone could act synergistically probably showing that insulin is required for growth through different mechanisms. Considering that growth is dependent of blood supply, we postulate that the role of insulin in growth implies the formation on new vessels by increasing the expression of VEGF. This may explain the occurrence of diabetic retinopathy with new vessels in presence of insulin and not with oral antidiabetic agents. The in vitro study of Meng et al. supports this hypothesis by pointing out the expression of VEGF in retinal microvascular endothelial cells as the hallmark in the pathophysiology of insulin-associated diabetic retinopathy [29]. They showed that insulin interacts with the NADPH oxidase subunit 4 (Nox-4) to induce excess production of reactive oxygen species (ROS) and subsequently oxidative stress in the retinal endothelial cells. ROS produced by insulin are involved in hypoxia-inducible factor-1α (HIF-1α) activation which in turn leads to the expression of VEGF. This latter mediates angiogenesis, neovascularization, and blood-retinal barrier disruption allowing vascular leakage as well [10, 29]. Considering these two findings of basic laboratory research, it appears that insulin is necessary for a growth to occur and is able to stimulate the growth of new vessels. Probably the worsening of diabetic retinopathy attributed to insulin use might result from ROS signaling via activation VEGF expression. This is a possible mechanism by which insulin administration can cause neovascularization as found in diabetic retinopathy.

## Evidence from epidemiological studies

Many epidemiological studies supported the fact that insulin therapy is a key factor in the occurrence of early worsening of diabetic retinopathy. To illustrate that, patients with type 1 diabetes who are almost exclusively treated with exogenous insulin are more prone to develop diabetic retinopathy after initiation of treatment as opposed to those with type 2 diabetes. For instance, at diagnosis of type 1 diabetes, most of patients do not have evidence of diabetic retinopathy. But, after 15 years of evolution and treatment of type 1 diabetes, over 90% of patients have evidence of diabetic retinopathy [3]. In comparison, at the diagnosis of type 2 diabetes (preclinical phase of up to 12 years), up to 20% of patients have evidence of diabetic retinopathy, and 15 years after the diagnosis (cumulated estimate of 27 years of evolution), only 60% of patients have evidence of diabetic retinopathy [3]. In addition, diabetic retinopathy is usually worse in adolescents and young adults compared to older individuals without any plausible explanation and the same findings have been reported in pregnancy [9]. This has long been associated with the natural course of the disease, but the possibility of an aggravation related to the treatment with insulin cannot be excluded. On the other hand, puberty and young adulthood are associated with intense growth of organs probably due to a surge of growth hormones. This hormonal surge coupled with exogenous insulin could synergistically induce neo-vessel formation in the eyes, based on the mechanism explained above. Moreover, patients with type 2 diabetes are older, are more likely to be treated with oral hypoglycemic agents, are more likely to have other risk factors of diabetic retinopathy, and are less likely to experience growth hormonal surge. The “synergistic effect” is thus less pronounced in patients with type 2 diabetes than in those with type 1 diabetes due to the less use of exogenous insulin for glucose lowering at least at the initiation of treatment. Despite the longer duration of type 2 diabetes and possible associated risk factors of diabetic retinopathy, the rate and severity of diabetic retinopathy is lesser than in type 1 diabetes. This could also explain the occurence of diabetic retinopathy found during pregnancy in some studies.

In a recent study, we reported and discussed the worsening of diabetic retinopathy observed after rapid lowering of blood glucose with insulin [2]. In a group of sub-Saharan African patients with type 2 diabetes who were screened for diabetic retinopathy using angiography, we observed that those treated with insulin alone had higher rates and more severe forms of diabetic retinopathy than those on oral agents alone [2]. This suggests that the “synergistic effect” is more pronounced in the group on insulin alone than in the group on oral anti-diabetic agents alone.

## Conclusions

The synergistic hypothesis appears to better explain the occurrence and worsening of diabetic retinopathy observed with the rapid lowering of blood glucose than the long held osmotic hypothesis, which only plausibly explained the occurrence of edema. This hypothesis has potential therapeutic implications as it could benefit patients with diabetes irrespective of the type. This suggests that, irrespective of the type of diabetes, the adjunction of an insulin sensitizer to any patient on insulin could be beneficial in terms of reduction of micro or macro-vascular complications. The dose of insulin needed to achieve blood glucose control will be less, thus a less pronounced synergy of insulin and VEGF. The hypothesis however needs to be studied in well-designed therapeutic trials.

## Abbreviations

DCCT: Diabetes Control and Complications Trial
HIF-1α: Hypoxia-inducible factor-1α
mEq: milli equivalent
NADPH: Nicotinamide Adenine Dinucleotide Phosphate
ROS: Reactive oxygen species
T2DM: Type 2 diabetes Mellitus
VEGF: Vascular Endothelial Growth Factor
